# Healthcare Professionals and Noise-Generating Tools: Challenging Assumptions about Hearing Loss Risk

**DOI:** 10.3390/ijerph20156520

**Published:** 2023-08-04

**Authors:** Giuseppe Alberti, Daniele Portelli, Cosimo Galletti

**Affiliations:** 1 Department of Adult and Development Age Human Pathology “Gaetano Barresi”, Unit of Otorhinolaryngology, University of Messina, 98125 Messina, Italy; galberti@unime.it; 2 Policlinico G. Martino, Via Consolare Valeria 1, 98125 Messina, Italy; 3 Department of Integrated Dentistry, School of Dentistry, Universitat Internacional de Catalunya, Sant Cugat del Vallès, 08017 Barcelona, Spain; cosimo88a@gmail.com

**Keywords:** noise-induced hearing loss, hidden hearing loss, ear surgeons, orthopaedic surgeons, dentists, dental hygienists, Matrix sentence test

## Abstract

Hearing loss is a significant global health concern, affecting billions of people and leading to various physical, mental, and social consequences. This paper focuses on the risk of noise-induced hearing loss (NIHL) among specific healthcare professionals, especially ear surgeons, orthopaedic surgeons, dentists, and dental hygienists, who frequently use noisy instruments in their professions. While studies on these professionals’ noise exposure levels are limited, certain conditions and factors could pose a risk to their hearing. Measures such as engineering and administrative controls, regular audiometric testing, and the use of hearing protection devices are crucial in preventing NIHL. Early detection and intervention are also vital to mitigate further damage. This paper proposes the results of a modified screening protocol, including questionnaires, audiometry, and additional diagnostic tests to identify and address potential hearing disorders. Specific healthcare professionals should remain aware of the risks, prioritize hearing protection, and undergo regular monitoring to safeguard their long-term auditory well-being.

## 1. Introduction

Hearing loss affects around 1.5 billion people globally, with the World Health Organization (WHO) predicting that by 2050, the number will reach nearly 2.5 billion [1]. Hearing loss has been linked to mental illness, depression, dementia, and social isolation, and it can also impact job performance, reduce productivity, and lead to work discrimination, unemployment, or loss of income [2]. Consequently, hearing loss has become a global public health priority. The leading causes of hearing loss are ageing, noise exposure, complications during birth, genetic factors, infectious diseases, chronic ear infections, or ototoxicity [1].

Occupational noise-induced hearing loss (NIHL) is a symmetrical sensorineural hearing loss that gradually develops after continuous or intermittent noise exposure. Following exposure to loud noise, a temporary threshold shift (TTS) or temporary hearing loss is present for 16 to 48 h. During the initial stages of NIHL, pure tone audiometry may appear normal, and there may be no observable signs of hearing loss on the hearing threshold. The condition referred to as “hidden hearing loss” is characterized by the damage or loss of synaptic connections between hair cells and cochlear neurons [3].

The gradual deterioration of auditory neurons is caused by this synaptopathy, which results in abnormal speech intelligibility and involvement of the hearing threshold at high frequencies (above 3 kHz) [4,5].

According to the Occupational Safety and Health Administration (OSHA), over 20 million workers are exposed to potentially harmful noise in the workplace every year [6]. Moreover, about 18% of adults aged 20–69 who were exposed to very loud noise at work for 5 or more years reported having NIHL [7]. As a result, various occupational and environmental noise exposure standards have been established worldwide [8] (see Table 1).

The OSHA and the European Agency for Safety and Health at Work have developed a hearing conservation program for individuals exposed to a noise level of 85 dBA or higher, averaged over 8 working hours [6,9]. The A-weighting (dBA) is used to measure sound levels, accounting for the loudness perceived by the human ear, which is less sensitive to low frequencies. This program aims to raise awareness among workers about the use of protective devices to prevent NIHL [6]. However, some individuals may be more susceptible to noise exposures between 80 and 85 dB [5]. Hearing impairment has a significant impact on daily life, with limited speech understanding and impaired communication. Additionally, tinnitus, headache, dizziness, or insomnia may be associated with hearing loss. The physical and psychological stress of hearing loss can reduce productivity, and increase the risk of workplace accidents and injuries.

The International Organization for Standardization (ISO) 1999:2013, titled “Acoustics—Estimation of noise-induced hearing loss”, provides comprehensive guidelines for assessing and estimating the impact of occupational noise exposure on individuals’ hearing health. The key objectives of ISO 1999:2013 include establishing criteria for classifying noise-induced hearing loss, defining audiometric evaluation procedures, calculating the risk of hearing impairment, and addressing permanent noise damage. It emphasizes the importance of considering factors such as ambient noise levels and exposure duration when evaluating the risk of hearing loss. Included within the standard are tables that present examples of predicted noise-induced permanent threshold shifts (NIPTS) at frequencies of 0.5, 1, 2, 3, 4, and 6 kHz. These predictions are based on L_EX8h_ exposures of 85, 90, 95, and 100 dBA, covering exposure durations ranging from 10 to 40 years. By utilizing these tables, it becomes feasible to calculate the average estimated NIPTS for the median of a population. According to the ISO 1999 standard, hearing impairment is defined with three definitions [10]:

- An average of the hearing threshold levels at 0.5, 1, and 2 kHz that exceeds 25 dB;
- An average of the hearing threshold levels at 1, 2, and 3 kHz that exceeds 25 dB;
- An average of the hearing threshold levels at 1, 2, 3, and 4 kHz that exceeds 25 dB.

It is essential to eliminate or minimize the risk of occupational NIHL. Early detection and intervention are crucial in preventing hearing loss, which is irreversible. General prevention principles can help reduce hearing impairment, such as adopting working methods or equipment that require less exposure to noise, using shields or noise-absorbing coverings, conducting periodic audiometric testing, and utilizing hearing protectors [6].

The demanding environment of the operating room presents specific healthcare professionals with numerous challenges, and one often overlooked aspect is the potential risk of noise-induced hearing loss. In the fast-paced world of operative procedures, professionals who use noise-generating tools rely heavily on a variety of instruments to perform intricate procedures. However, the use of noisy instruments poses a unique challenge. Prolonged exposure to high levels of noise during these procedures may have detrimental effects on the professionals’ hearing abilities.

This paper provides a concise overview of the hearing risks faced by some specific healthcare workers who are not considered at high risk of developing occupational NIHL.

Noise exposure levels have been recorded in various operating rooms and reported in different studies with contrasting results. The levels of noise can vary significantly depending on several factors. The design and layout of the theatres, the types of equipment and instruments utilized, as well as the procedures being performed, all contribute to the overall noise levels experienced by surgical teams [11].

Our focus is on four categories of professionals, including ear surgeons, orthopaedic surgeons, dentists, and dental hygienists, specialities that involve the use of noisy tools.

Otorhinolaryngologists, specifically ear surgeons, may face high levels of noise exposure during temporal bone drilling. Although few studies have measured the risk in the operating room, Vaisbuch et al. researched noise exposure during temporal bone drilling, even when multiple individuals were drilling simultaneously in dissection labs [4]. Some studies have reported exposure levels ranging from 68.5 to 83 dBA over an 8-h period, which did not surpass the limits established by the OSHA [12,13]. However, certain drilling conditions and environmental factors could pose a particular risk to workers’ hearing. Factors such as the type of drill (air or piezoelectric), the burr type (burr diameter, cutting or diamond burr), the anatomical structure being drilled (cortical bone or mastoid cavity), and the surgical approach (translabyrinthine surgery, middle ear only, or mastoid surgery) could produce peak sound pressure levels that exceed the safe limit. Additionally, the frequency range to which the worker is exposed could vary based on these variables [4].

Orthopaedic surgeons use various power tools and loud instruments such as drills, saws, and hammers during surgery [11,14]. However, few studies have been conducted to assess noise levels in orthopaedic surgery. In a study by Goffin et al., the equivalent continuous sound pressure levels were recorded during elective arthroplasty (total hip replacement and total knee replacement), but the personal noise exposure did not reach the 80 dB daily A-weighted noise exposure level [11]. On the other hand, Mullett’s study revealed that most of the cited instruments generated sound pressure levels above 90 dB, increasing the risk of premature hearing loss development. A recent study showed that noise levels reached 105.6 dBA when using a hammer and 97.9 dBA when using an oscillating saw; in these latter cases, measurements were taken considering A-weighted decibel levels, recorded with fast time weighting, once per second. [15]. However, the modernity of these tools could influence the noise level, as new instruments can generate less noise than older ones [16]. While brief and intermittent bursts of intense noises during orthopaedic surgery may reduce the risk of hearing loss or tinnitus development, further studies are necessary to confirm the association between orthopaedic occupational exposure and NIHL [11,14]. Nonetheless, personnel working in orthopaedic theatres should be aware of the potential long-term health hazard [17].

Occupational noise that may be hazardous to dental practitioners and hygienists and has been recorded even in dental healthcare settings [18,19,20]. Professionals in this field are exposed to noise produced by air-turbine or micromotor handpieces, ultrasonic scalers, suction tubes, and laboratory equipment. Studies have measured and reported the noise levels of these instruments, with clinical handpieces ranging from 76 to 105 dBA, suction ranging from 74 to 80 dBA, and cleaners and scalers ranging from 82 to 90 dBA [20,21,22,23,24]. The literature reports high variability in these noise levels, which may be influenced by various factors such as the duration of noise exposure, the type of dental speciality, and the use of faulty or worn equipment, particularly turbines [18,19]. Burk et al. have suggested that a small portion of dental professionals and students may be at risk of developing NIHL despite the reported mean 8-h time-weighted average (TWA) noise levels of 70.9 dBA [20]. Ultrasonic scalers, which produce a sound pressure level ranging from 87 to 107 dBA have also received attention as potential causes of temporary shifts in the hearing threshold, tinnitus, and a statistically significant difference in the audiometric threshold at the frequency of 3000 Hz. It should be noted, however, that these instruments generate sounds at ultra-high frequencies that are inaudible to humans regardless of intensity [25,26]. It is important to highlight that the majority of these measurements were brief recordings conducted in close proximity to operating dental instruments, rather than assessing personal exposure. Additionally, it should be noted that the A-weighting network, commonly used for occupational exposure assessments, does not fully capture noise exposures related to ultrasonic devices, as it heavily attenuates ultrasonic frequencies (>20 kHz) [20].

However, based on our experience and case series, it is not common for practitioners in these fields to utilize hearing protection devices. This lack of protection leaves them vulnerable to exposure to loud and potentially harmful levels of noise in their work environment [27].

To prevent the progression of auditory damage, early detection of these conditions is essential for individuals who are exposed to loud sounds. We firmly believe that regular audiological screening is necessary for such individuals to detect a hearing impairment.

**Figure 1:**
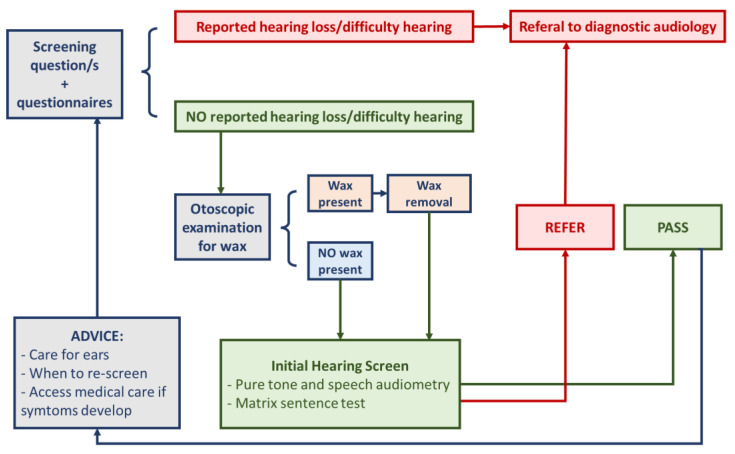
Modified WHO adult hearing screening (Adapted from: Hearing screening. Consideration for implementation. WHO. 2021 [28]).

Below, we outline a modified adult screening procedure recommended by the World Health Organization, which is followed at our tertiary referral centre, Policlinico “G. Martino” in Messina (Italy) [28] (see Figure 1). Due to the lack of validation of our screening protocol, the full substantiation of its effectiveness remains inconclusive. However, it may hold potential as a valuable tool in clinical practice, aiding in early identification of potential hearing issues for timely intervention and improved patient outcomes. Furthermore, we present a retrospective analysis of data from the past 2 years, obtained from 42 people who underwent screening.

## 2. Materials and Methods

### 2.1. Participants

A total of 42 individuals, comprising ear surgeons (10 subjects), orthopaedic surgeons (14 subjects), dentists, and dental hygienists (18 subjects), underwent a hearing screening (see Figure 1). These participants were selected based on specific inclusion criteria, which included a minimum of 10 years of occupational experience in their respective fields and consistent exposure to noise-emitting tools during this period. All subjects did not use any type of noise attenuator or hearing protector. They also had no pre-existing diagnosed conditions or were not on chronic medication therapies for any specific medical conditions. Subjects were invited to undergo free hearing screening through online advertisements, word-of-mouth among acquaintances, or direct invitations. All tests were conducted at Policlinico “G. Martino” in Messina, a tertiary referral centre. The data presented here are reflective of the past two years.

### 2.2. Screening Questions and Questionnaires

Simple anamnestic questions regarding hearing are employed as part of the screening process. These questions provide a comprehensive overview of the individual’s hearing health and help direct the screening investigation. A combination of binary (Yes/No) response questions and scored questions are utilized (see Table 2).

These questions allow for quick assessments and are designed to capture specific aspects of the individual’s hearing health. They cover topics such as exposure to loud noise, history of ear infections or trauma, and the use of hearing protection devices. These questions enable healthcare professionals to identify potential risk factors and determine the need for further evaluation.

Furthermore, validated questionnaires can be employed to delve deeper into the initial investigation. These questionnaires have been extensively tested and proven to be reliable tools for assessing specific aspects of hearing. By utilizing these validated questionnaires, healthcare professionals can gather more comprehensive data and gain further insights into the individual’s hearing health status.

Typically, we employ three questionnaires:

1. The Self-Efficacy for Situational Communication Management Questionnaire (SESMQ), which measures the perceived self-efficacy for managing communication in individuals with hearing loss. The SESMQ questionnaire consists of 20 questions, each divided into two scales, and the subject is required to respond by assigning a score from 1 to 10. The first scale assesses hearing ability (SESMQH), while the second scale evaluates the level of confidence (SESMQ). Each subscale has a score ranging from 10 to 200. The total score is the sum of the two subscale scores [29];
2. The Noise Exposure Questionnaire (NEQ), which quantifies personal annual noise exposure from both occupational and non-occupational sources. The NEQ questionnaire consists of three sections: the first section includes demographic information, the second section contains six screening questions that assess exposure to high levels of noise, and the third section consists of eleven questions related to participation in noisy activities, on which the annual exposure level is calculated. Three questions in the second section (1-min Noise Screen) serve as a screening tool to assess exposure to high levels of noise, with a score greater than 4 indicating a high risk of developing NIHL (Noise-Induced Hearing Loss). The NEQ questionnaire calculates the annual noise exposure, and the risk of developing NIHL occurs when the L_Aeq8760h_ (annual equivalent sound level) is equal to or greater than 79 dB [30];
3. The Hearing Handicap Inventory for the Elderly (HHIE), which assesses the impact of hearing impairment on emotional and social adjustment. The HHIE questionnaire consists of 25 questions. The individual assigns a score ranging from “yes” (4 points), “sometimes” (2 points), to “no” (0 points). The total score ranges from 0 to 100 [31].

### 2.3. Audiological Screening Tests

Pure-tone audiometry, speech audiometry, and the free-field Matrix sentence test are used as audiological screening tests. These are conducted within a soundproof booth that attenuated environmental noise by 40 dB SPL. The tests are performed using the Madsen Astera2 audiometer (Otosuite V. 8.84.0 software, Taastrup, Denmark), which had been calibrated within the last 12 months prior to the measurements. For the audiometric evaluations, TDH39 earphones, a B71 bone vibrator, and speakers are utilized.

Pure-tone audiometry is employed to determine the individual’s hearing threshold. Speech audiometry is also conducted using spondaic disyllabic Italian words [32]. The word recognition score (WRS) is measured as the percentage of correctly recognized words when a list of ten words is presented to the individual. No masking noise is used during this test. The air conduction pure-tone average (AC PTA) and the bone conduction pure-tone average (BC PTA) are automatically computed based on the hearing thresholds at 500, 1000, and 2000 Hz; furthermore, a modified air conduction pure-tone average (AC mPTA) and a modified bone conduction pure-tone average (BC mPTA) considering the frequencies 1000, 2000, and 4000 Hz are utilized, as suggested by Moore et al., to better assess noise-induced hearing loss. This modified PTA approach allows for a more focused evaluation of the specific frequency range commonly affected by noise-induced hearing impairments [33].

To assess speech-in-noise intelligibility, the Italian Matrix sentence test is performed. For this test, two speakers are positioned at a distance of 1 m from the participant, with one on the left side and the other on the right side (azimuth angles of −45° and +45°, respectively). This is an adaptive speech-in-noise test that consists of 20 randomly generated sentences, each composed of five words, with a background noise interfering with the speech message. The subject is asked to repeat each word they can hear [34]. If the subject accurately repeats at least three words, the speech level is decreased; otherwise, it is increased. The Matrix software computes the speech reception threshold (SRT), which represents the 50% threshold of speech intelligibility in noise (dB SNR). This test is valuable for evaluating both the individual’s ability to recognize speech in noisy environments and the effectiveness of hearing aids [35,36].

The purpose of the audiological screening tests is to detect the possible presence of noise-induced hearing loss or other disorders in the auditory system such as tinnitus or abnormal speech intelligibility. Noise-induced hearing loss (NIHL) is a symmetrical sensorineural hearing loss that gradually develops after continuous or intermittent noise exposure [5].

### 2.4. Procedure

All subjects underwent an anamnestic questionnaire (see Table 2) to assess their hearing health status. These questions were designed to guide subsequent screening steps. All subjects were administered the SESMQ questionnaire and the 1-min Noise Screen questionnaire. It should be noted that the SESMQ questionnaire was initially developed for elderly subjects with acquired hearing loss, and its validity in younger individuals has not yet been established [29]. However, this questionnaire can provide an estimate of the subjectively perceived level of disability in communication ability. A score of 5 or higher on the 1-min Noise Screen indicates a high risk of noise exposure, and thus, these subjects were further evaluated with the comprehensive NEQ questionnaire. Subjects who perceived a decrease in their hearing abilities or tinnitus were also administered the HHIE questionnaire.

When questionable results were obtained, an audiological assessment was strongly recommended. For individuals who do not report hearing problems, a second screening test was performed.

Subsequently, all subjects underwent otomicroscopic examination to assess the presence of earwax, which was removed if necessary. Following this, pure-tone audiometry, speech audiometry with headphones, and the free-field Matrix Sentence Test were performed.

“PASS” indicates a condition in which there is no alteration in the audiometric threshold or speech-in-noise intelligibility, as determined by the results of the Matrix sentence test. On the other hand, “REFER” indicates an alteration in one or both of the audiologic tests used.

Individuals who receive a “PASS” result in these hearing screening tests will undergo another screening after about two years. Those who receive a “REFER” result will require further evaluation for the presence of any “red flags” alerts, such as rapidly progressive hearing loss, unilateral hearing loss, ear pain, ear discharge, dizziness, or previous diagnosis of ear disease [28].

Doubtful results in these screening tests required more accurate diagnostic approaches, such as auditory brainstem response (ABR), otoacoustic emissions (OAE), magnetic resonance imaging (MRI) of the brain and brainstem, and computed tomography (CT), tympanometry and acoustic reflex testing, etc. Furthermore, overlapping pathological conditions may be present in the patients, and they should not be underestimated.

#### Results

In our case series, a total of 42 subjects (27 males and 15 females) underwent screening. Among them, 10 were ear surgeons, 14 were orthopaedic surgeons, and 18 were dentists or dental hygienists. The average age of these subjects was 48.5 ± 9.5 years, with an average work experience of 22.1 ± 9.5 years (see Table 3).

Analysing the data obtained from the audiometric thresholds, the mean of AC mPTA for the right ear was 19.2 ± 7.6 dB HL, and for the left ear, it was 19.4 ± 7.2 dB HL. As for the word recognition score (WRS), all subjects achieved the 100% intelligibility threshold.

Regarding the speech reception threshold (SRT), the average was −6.3 ± 0.9 dB SNR. Two subjects recorded an increased SRT despite having a normal hearing threshold (−3.6 dB SNR, −4.8 dB SNR). These values contradicted the normative data presented by Puglisi et al. for the Italian version of the Matrix sentence test [34].

The subjects were then stratified into two groups based on the presence or absence of a hearing handicap. A “hearing handicap” is defined as an impairment in the auditory system that can be objectively measured through the used screening audiological tests or subjectively reported by the person from the anamnestic questions and questionnaires for screening, such as tinnitus or decreased speech-in-noise intelligibility. Thirteen subjects showed evidence of hearing issues: high-frequency notch at 4 kHz (four subjects), otosclerosis (one subject), high-frequency hearing loss (three subjects), tinnitus (three subjects), and elevated SRT with normal hearing threshold (two subjects) (see Table 4 and Table 5).

From the screening anamnestic questions (questions “j” and “k”, see Table 2), it was observed that all subjects lacked awareness of the noise they were exposed to, considering it insufficiently intense to cause noise-induced hearing loss. Additionally, from a subjective perspective, four subjects without a hearing handicap and three subjects with a hearing handicap reported experiencing a sensation of muffled hearing immediately following the cessation of exposure to the noisy instrument. All subjects reported that this sensation lasted between 5 and 15 s and disappeared thereafter. We hypothesize that this phenomenon may be attributed to temporary auditory fatigue.

The average total score of the SESMQ questionnaire was 326.2 ± 32.4. The questionnaire revealed lower scores in subjects with hearing problems (see Table 6).

On the other hand, the 1-min Noise Screen allowed the identification of individuals at higher risk of developing noise-induced hearing loss, considering both recreational and job activities. The average score obtained was 3.3 ± 1.4, indicating that these subjects were not at a high risk of noise-induced hearing loss (see Table 7). It should be noted that this is the average score among all participants. However, out of the 42 participants, 8 obtained scores higher than 4, and thus, they underwent the complete NEQ questionnaire. Only two of them demonstrated annual L_Aeq8760h_ (annual equivalent sound level) exposure levels exceeding 79 dB, indicating a risk of developing hearing loss.

Regarding the HHIE questionnaire, the test was administered to the 13 subjects who reported a hearing handicap; the average score was 17.4 ± 11. Only subjects who had a hearing handicap were administered this test; no other subjects reported any hearing problems.

We want to clarify that no statistical analysis has been conducted as the presented article takes the form of an “opinion” rather than a research study aimed at demonstrating the association between the use of noise-emitting tools and noise-induced hearing loss or even other hearing disorders. The screening protocol is currently undergoing validation, and the results presented are only preliminary data. Subjects were stratified solely for descriptive purposes and not for comparison. The literature contains variable data regarding exposure levels, making it unable to definitively state the absence of a real risk for these individuals. Therefore, the possibility of developing noise-induced hearing loss or other auditory issues should always be taken into consideration.

## 3. Discussion

The best way to reduce the risk of developing occupational NIHL is through prevention measures, as the condition is irreversible and there are currently no effective treatments available. The prevention program is based on three levels: primary prevention, which involves reducing noise exposure levels below hazardous levels; secondary prevention, which focuses on early detection of hearing loss to prevent further damage; and tertiary prevention, which aims to reduce disability or handicap when significant impairment is already present [9,37].

The European Agency for Safety and Health at Work emphasizes the importance of maintaining a safe and healthy work environment, including periodic monitoring of noise exposure. When high levels of noise are detected, engineering and administrative controls are necessary to prevent irreversible damage to the inner ear. These controls include: (1) selecting appropriate work equipment; (2) providing adequate information and training to ensure proper use of work equipment; (3) reducing noise levels through technical means; (4) implementing maintenance programs for work equipment, workplace, and workplace systems; (5) organizing work to minimize noise levels; (6) limiting the duration and intensity of noise exposure; (7) establishing work schedules that include adequate rest periods [9].

The risk of developing occupational NIHL can be significantly reduced or eliminated when the noise level is kept below 80 dBA. Legal standards for noise levels have been established by various countries [8]. When noise levels cannot be reduced, workers should use hearing protection devices. Two types of hearing protection devices are currently available: active and passive devices. Research has demonstrated that these devices are effective at reducing sound levels, allowing workers to locate the source of sounds, and enabling communication [38].

Elevated noise levels have been observed to have a detrimental impact on patient outcomes and impair the performance of healthcare professionals in the operating room. Despite the increase in decibel levels caused by playing music in the operating room, the majority of surveyed staff expressed a positive inclination towards having music during surgery, believing it to enhance both individual and team performance. Overall, music was not perceived as a distraction or hindrance to communication [39]. Some studies indicated a notable decrease in postoperative complication rates when noise levels were reduced during surgery [40,41,42]. In a study conducted in a paediatric surgery department, various measures were implemented to reduce noise levels, including the use of sound-reduction devices, behavioural guidelines restricting conversation, minimizing the opening of operating room doors, and managing monitor alarms. This comprehensive noise reduction program resulted in a significant reduction of approximately 50% in decibel levels during paediatric surgical procedures [42].

Other measures of well-being could be applied in the environments where operative procedures are conducted. In a study that proposed the revitalization of a park, it was highlighted that it is not only necessary to reduce noise exposure levels or introduce positive sounds, but also to implement measures that enhance overall well-being. A similar approach could be employed in the workplaces where the subjects under consideration operate. Further investigation is needed to explore the most effective measures, making this area a promising research field yet to be fully explored [43].

Secondary prevention is achieved through regular audiometric testing. Implementing regular auditory assessments and monitoring programs can help detect any early signs of hearing loss in professionals who use noise-generating tools. Prompt identification of hearing impairment allows for timely intervention and the implementation of strategies to mitigate further damage.

However, there is still some uncertainty in the literature regarding noise exposure for these subjects who work with noisy instruments. Although these workers are not typically considered at high risk for developing occupational NIHL, it has been reported that noises below the action level can cause temporary and potentially long-term hearing damage [4].

Animal studies have shown that moderate noise exposure (100 dB SPL for 2 h) can cause a TTS without resulting in hair cell loss. However, repeated TTSs, even with threshold recovery, can alter cochlear responses to suprathreshold sound levels. This can result in a synaptopathy, which is characterized by a reduced number of auditory nerve fibres’ activation and a decrease in their firing rate or synchrony. This condition is known as “hidden hearing loss” and is characterized by a normal hearing threshold but difficulties in complex listening tasks, such as word recognition, accurate speech, and sound detection in noisy environments [44].

According to Moore et al., pure tone and speech audiometry are valuable tests in diagnosing NIHL. NIHL caused by different types of noises can be distinguished. Additionally, it is advisable to compare the patient’s hearing threshold with the age-related hearing levels for a non-noise-exposed population, typically using the 50th percentile as specified in ISO 7029 (2017). The NIHL should be quantified for each ear by considering the mean hearing threshold across the frequencies of 1, 2, and 4 kHz [33].

The diagnosis of hidden hearing loss warrants a separate discussion. Kohrman et al. have described three diagnostic approaches for this. The first approach involves the auditory brainstem response (ABR), which shows a reduction in the amplitude of the ABR I peak without any changes in ABR threshold or latency. This reduction correlates with the degree of cochlear synaptopathy. The second approach involves frequency following responses (FFRs), which demonstrate a decline in modulation frequency near 1 kHz, correlating with synaptic loss. The third approach involves a weaker middle ear muscle reflex response in individuals with hidden hearing loss [44].

In this study, we presented the data related to the screening protocol followed at our tertiary referral centre. It is important to note that these are preliminary findings. The screening program has been ongoing for two years, and concrete data on subject follow-up are not yet available.

As mentioned earlier, the subjects were recruited among ear surgeons, orthopaedic surgeons, dentists, and dental hygienists. We deemed it appropriate to select only subjects with more than 10 years of experience, as the risk of noise-induced hearing loss increases with prolonged exposure.

As outlined in the Materials and Methods section, the initial step involved collecting subject histories to guide the clinician in the appropriate screening pathway. The SESMQ questionnaire and the 1-min Noise Screen were administered to all subjects, while the HHIE questionnaire was given only to those who reported subjective hearing loss or a hearing handicap. It is important to note that these questionnaires are easy and reliable tools for initial diagnostic assessment but should be integrated with objective tests. Questionnaires provide subjective estimates in a rapid manner, as they can be completed in a few minutes. They also have the advantage of being sent via email or filled in online, allowing for quick data collection and early identification of individuals at risk of hearing loss. However, it should be noted that the 1-min Noise Screen may not identify subjects at risk of noise-induced hearing loss since these people may not perceive their noise exposure as risky. This may necessitate the administration of the full NEQ questionnaire.

Regarding the administration of the HHIE questionnaire, in our protocol, it is given only to subjects who perceive hearing loss. In this case, as well, biases may occur as some subjects may perceive hearing loss despite having normal auditory function (not in our case series).

Furthermore, questionnaires are tools that should be used to rapidly obtain an effective estimation of the risk of noise-induced hearing loss or deafness. Therefore, a streamlined procedure must be applied when extending the screening protocol to many subjects to ensure compliance.

However, in our protocol, all subjects subsequently undergo more reliable tests such as pure-tone audiometry, speech audiometry, and the Matrix sentence test. Subjects with normal results, indicating a PASS, are rescreened after 2 years. Those with hearing problems undergo more comprehensive examinations.

In our case series of 42 subjects, 13 individuals demonstrated a hearing handicap: these were referred for further instrumental examinations. The data for these patients are currently unavailable as the diagnostic assessment is still ongoing; hence, concrete results cannot be presented at this time.

Out of the eight subjects who completed the full NEQ questionnaire, only two subjects exceeded an annual L_Aeq8760h_ of 79 dB: one subject with a notch at 4 kHz and one subject without hearing impairment.

It is important to reiterate that the data presented in this study are preliminary. The hearing problems observed in these subjects cannot be definitively attributed to noise-induced damage: modifiable risk factors (smoking, alcohol, obesity, etc.) and non-modifiable factors (age, sex, genetics), as well as comorbidities or ototoxic drugs, can influence the possibility of hearing damage. The purpose of this study is not to establish a causal relationship between the “risk factor” of using noise-emitting tools and the “disease” itself, namely noise-induced hearing loss, but rather to raise awareness about the potential impact of the work environments and tools utilized on the individuals’ auditory system. Establishing a potential correlation between noise-induced hearing loss in the considered healthcare workers requires appropriate studies. The noise exposure levels to which the subjects are exposed, necessary according to the ISO 1999–2013 standard to assess the risk of developing noise-induced hearing loss, were not considered in this paper, as the focus is to describe a possible streamlined and rapid screening program. Subjects may be exposed to varying levels of noise, and it is uncertain whether some of these levels are of sufficient intensity to cause auditory damage. Measuring exposure levels requires specific protocols that are challenging to implement in a screening program. However, the purpose of this analysis is to draw attention to a relevant public health issue. We are convinced that the conflicting results present in the literature require further confirmation to avoid underestimating the risk faced by these individuals and to identify the most appropriate screening protocol. Due to the presence of numerous limitations and biases in our work, as a future perspective, and an expansion of the scientific literature, a more refined and validated screening program should be developed. Studies demonstrating the association between noise-emitting operative tools and noise-induced hearing loss, or other hearing impairments, should be conducted, considering the execution of randomized clinical trials with a larger sample size or multicenter studies. Additionally, evaluating noise exposure levels and taking into account human exposure to noise, considering the position of the operator’s head and body, would provide more accurate measurements. Therefore, a binaural measurement approach would be recommended.

## 4. Conclusions

Noise-induced hearing loss is a real occupational hazard faced by surgeons and other healthcare professionals due to prolonged exposure to high levels of noise in the operating room. Preventing occupational noise-induced hearing loss is crucial as the condition is irreversible and currently has no cure. To achieve this goal, a prevention program is recommended.

Creating awareness among professionals who use noise-generating tools about the importance of noise management in the operating room is essential. Education and training programs can emphasize the significance of adopting noise reduction strategies while using these instruments when necessary.

Despite being exposed to high noise levels, these subjects may not be considered a high-risk group for developing hearing handicaps. Factors such as the short duration of exposure, distance from noise sources, awareness of the risks, and regular auditory assessments contribute to the preservation of their hearing health.

The variability in noise levels underscores the importance of conducting individualized assessments for each operating room. Understanding the specific factors that contribute to noise generation in each specialty can aid in implementing appropriate noise control measures.

From the data we collected, out of the 42 subjects analysed, 13 individuals had a hearing handicap. Among these, one case was attributed to otosclerosis and not related to noise exposure. As for the remaining 12 subjects, it cannot be definitively stated that their hearing handicap is solely attributable to noise exposure, but it cannot be ruled out either. These individuals exhibit deficits compatible with noise-induced damage. While we acknowledge that our screening currently lacks validation, the analysis of data from these subjects still revealed the presence of some individuals with a hearing handicap.

In this article, we aim to advocate for the importance of regular screenings in these individuals, as there is a lack of awareness, based on our experience, regarding the potential risk of developing hearing impairment. Although the association has not been proven, screening can serve as an early diagnostic tool for identifying potential hearing deficits among these subjects.

If a potential association between noise-induced hearing loss and healthcare workers who use noisy instruments is confirmed, efforts should be directed towards optimizing the acoustic environment in operating rooms through the use of sound-absorbing materials, proper room design, and the adoption of quieter surgical instruments where feasible. Additionally, the use of personal protective equipment, such as noise attenuators or hearing protectors, should be considered to minimize the risk of prolonged exposure to high noise levels.

We believe that further research and collaboration between healthcare professionals, audiologists, and equipment manufacturers are needed to establish standardized guidelines and best practices for noise control in operating rooms. By addressing the variability in noise levels across different specialties and implementing appropriate mitigation strategies, the overall work environment can be made safer and more conducive to optimal surgical outcomes and the well-being of surgical teams.

However, it is crucial for these workers to remain vigilant, prioritize hearing protection, and continue to monitor their hearing to ensure long-term auditory well-being, protecting the “gift of sound”.

## Figures and Tables

**Table 1:** Examples of occupational noise exposure limits (adapted from: Neitzel, 2019).

Category/Limit	Allowable Exposure (dBA) for Given Time Period				
	8 h	2 h	1 h	30 min	Ceiling
European Union Directive 2003/10/EC (European Parliament and Council, 2003)					
Lower exposure action value	80	83	86	89	//
Upper exposure action value	85	88	91	94	//
Exposure limit	87	90	93	97	//
American Conference of Governmental Industrial Hygienists (ACGIH, 2018a)					
Threshold limit value	85	88	91	94	//
US National Institute for Occupational Safety and Health (NIOSH, 1998)					
Recommended exposure limit	85	88	91	94	//
U.S. Occupational Safety and Health Administration (OSHA, 1983)					
Permissible exposure limit	90	95	100	105	115
Action level	85	90	95	100	115

Note: “//”, Allowable exposure dBA not provided.

**Table 2:** Examples of questions and questionnaires for screening (Adapted from Hearing screening. Consideration for implementation. WHO. 2021 [28]).

Screening Questions and Questionnaires
A. Yes/No questions: a. Do you have any hearing problem now? b. Do you have a diagnosed hearing loss? c. Do you use hearing aids? d. Have you noticed any changes in your hearing abilities recently? e. Do you experience difficulty understanding conversations in noisy environments? f. Have you ever had any exposure to loud noise, either in your personal or professional life? g. Have you ever experienced ringing or buzzing sounds in your ears (tinnitus)? h. Are there any instances where you struggle to hear certain frequencies or sounds? i. Have you ever had your hearing tested or undergone any auditory assessments? j. Are you aware of the risks associated with noise-induced hearing loss? k. Have you noticed any impact on your hearing after performing the procedures or being in the operating room? B. Scaled questions: a. How would you characterize your hearing? i. Excellent ii. Very good iii. Good iv. Fair v. Poor C. Existing screening questionnaire a. Self-Efficacy for Situational Communication Management Questionnaire (SESMQ) b. Noise Exposure Questionnaire (NEQ) and 1-min Noise Screen c. Hearing Handicap Inventory for the Elderly (HHIE)

**Table 3:** Study population. N, number; %, percentage; M, mean; SD, standard deviation; AC PTA, air conduction pure tone average; AC mPTA, air conduction modified pure tone average; BC PTA, bone conduction pure tone average; BC mPTA, bone conduction modified pure tone average; WRS, word recognition score; SRT, speech reception threshold; SNR, signal to noise ratio.

	N (%); M ± SD
Gender Male Female	42 (100%) 27 (64%) 15 (36%)
Profession Ear surgeons Orthopaedic surgeons Dentists and dental hygienists	42 (100%) 10 (24%) 14 (33%) 18 (43%)
Age (years)	48.5 ± 9.5
Worked years (years)	22 ± 9.5
AC PTA Right (dB HL)	17.1 ± 5.5
AC mPTA Right (dB HL)	19.2 ± 7.6
AC PTA Left (dB HL)	18.0 ± 5.5
AC mPTA Left (dB HL)	19.4 ± 7.2
BC PTA Right (dB HL)	11.9 ± 5.2
BC mPTA Right (dB HL)	14.0 ± 7.5
BC PTA Left (dB HL)	12.6 ±4.7
BC mPTA Left (dB HL)	14.0 ± 6.8
WRS Right % (dB HL)	100 ± 0 (40.7 ± 7.1)
WRS Left % (dB HL)	100 ± 0 (40.7 ± 7.1)
SRT (dB SNR)	−6.2 ± 0.9

**Table 4:** Number of subjects with and without hearing handicap. N, number; %, percentage; SRT, speech reception threshold.

Subjects with	N (%)
No hearing handicap	29 (69%)
Hearing handicap 4 kHz notch on hearing threshold Otosclerosis High frequencies hearing loss Tinnitus Increased SRT with normal hearing threshold	4 (9.5%) 1 (2.4%) 3 (7.1%) 3 (7.1%) 2 (4.8%)

**Table 5:** Subjects’ data obtained from audiological screening tests. M, mean; SD, standard deviation AC PTA, air conduction pure tone average; AC mPTA, air conduction modified pure tone average; SRT, speech reception threshold.

Subjects with	AC PTA (Right; Left) (M ± SD)	AC mPTA (Right; Left) (M ± SD)	SRT (M ± SD)
No hearing handicap	14.6 ± 3.2; 15.7 ± 2.6	15.3 ± 3; 15.8 ± 2.4	−6.7 ± 0.5
Hearing handicap 4 kHz notch on hearing threshold Otosclerosis High frequencies hearing loss Tinnitus Increased SRT with normal hearing threshold	21.3 ± 3.5; 20.5 ± 5.8 25; 35 * 29.7 ± 2.5; 30 ± 2; 21.3 ± 5.1; 20.7 ± 3.2 17.0 ± 0; 16.5 ± 2.1	29.3 ± 2.9; 27.3 ± 4.3 23; 32 * 38.7 ± 2.9; 38.3 ± 2.9 23.3 ± 4.2; 22.3 ± 0.6 17.5 ± 0.7; 17.5 ± 0.7	−5.2 ± 0.4 −5.2 * −5.4 ± 0.3 −5.6 ± 0.2 −4.2 ± 0.8

* The AC PTA, the AC mPTA, and the SRT pertain to a single case only; data are presented as mean.

**Table 6:** SESMQ questionnaire results. M, mean; SD, standard deviation; SESMQH, Self-Efficacy for Situational Communication Management Questionnaire Hearing ability score; SESMQC, Self-Efficacy for Situational Communication Management Questionnaire Confidence score; SESMQ, Self-Efficacy for Situational Communication Management Questionnaire global score.

Subjects with (N)	SESMQH (M ± SD)	SESMQC (M ± SD)	SESMQ (M ± SD)
No hearing handicap (29)	175.9 ± 5.3	166.0 ± 6.2	341.9 ± 10.9
Hearing handicap (13)	148.2 ± 17.9	142.9 ± 19.9	291.1 ± 37.2
Total of subjects (42)	167.4 ± 16.8	158.8 ± 16.1	326.2 ± 32.4

**Table 7:** 1-min Noise Screen results. M, mean; SD, standard deviation.

Subjects with	1 min Noise Screen (M ± SD)
No hearing handicap	3.0 ± 1.3
Hearing handicap	4.1 ± 1.3
Total of subjects	3.3 ± 1.4

## Data Availability

The data presented in this study are available on request to the corresponding author. The data are not publicly available as they may contain sensitive patient information, including medical history, questionnaire results (which may include personal information), and audiological test results.
